# Antibacterial and Herbicidal Activity of Ring-Substituted 2-Hydroxynaphthalene-1-carboxanilides

**DOI:** 10.3390/molecules18089397

**Published:** 2013-08-06

**Authors:** Tomas Gonec, Jiri Kos, Iveta Zadrazilova, Matus Pesko, Rodney Govender, Stanislava Keltosova, Barbara Chambel, Diogo Pereira, Peter Kollar, Ales Imramovsky, Jim O’Mahony, Aidan Coffey, Alois Cizek, Katarina Kralova, Josef Jampilek

**Affiliations:** 1 Department of Chemical Drugs, Faculty of Pharmacy, University of Veterinary and Pharmaceutical Sciences, Palackeho 1/3, 612 42 Brno, Czech Republic; 2 Department of Infectious Diseases and Microbiology, Faculty of Veterinary Medicine, University of Veterinary and Pharmaceutical Sciences, Palackeho 1/3, 612 42 Brno, Czech Republic; 3 CEITEC VFU, University of Veterinary and Pharmaceutical Sciences, Palackeho 1/3, 612 42 Brno, Czech Republic; 4 Department of Environmental Ecology, Faculty of Natural Sciences, Comenius University, Mlynska dolina Ch-2, 842 15 Bratislava, Slovakia; 5 Department of Biological Sciences, Cork Institute of Technology, Bishopstown, Cork, Ireland; 6 Department of Human Pharmacology and Toxicology, Faculty of Pharmacy, University of Veterinary and Pharmaceutical Sciences, Palackeho 1/3, 612 42 Brno, Czech Republic; 7 Institute of Organic Chemistry and Technology, Faculty of Chemical Technology, University of Pardubice, Studentska 573, 532 10 Pardubice, Czech Republic; 8 Institute of Chemistry, Faculty of Natural Sciences, Comenius University, Mlynska dolina Ch-2, 842 15 Bratislava, Slovakia

**Keywords:** hydroxynaphthalenecarboxanilides, lipophilicity, photosynthetic electron transport inhibition, spinach chloroplasts, *in vitro* antibacterial activity, *in vitro* antimycobacterial activity, *in vitro* cytotoxicity, structure-activity relationships

## Abstract

In this study, a series of twenty-two ring-substituted 2-hydroxynaphthalene-1-carboxanilides were prepared and characterized. Primary *in vitro* screening of the synthesized compounds was performed against *Staphylococcus aureus*, three methicillin-resistant *S. aureus* strains, *Mycobacterium marinum*, *M. kasasii*, *M. smegmatis*. and *M. avium paratuberculosis.* The compounds were also tested for their activity related to inhibition of photosynthetic electron transport (PET) in spinach (*Spinacia oleracea* L.) chloroplasts. 2-Hydroxy-*N*-phenylnaphthalene-1-carboxanilide and 2-hydroxy-*N*-(3-trifluoromethylphenyl)naphthalene-1-carboxamide (IC_50_ = 29 µmol/L) were the most active PET inhibitors. Some of tested compounds showed the antibacterial and antimycobacterial activity against the tested strains comparable or higher than the standards ampicillin or isoniazid. Thus, for example, 2-hydroxy-*N*-(3-nitrophenyl)naphthalene-1-carboxamide showed MIC = 26.0 µmol/L against methicillin-resistant *S. aureus* and MIC = 51.9 µmol/L against *M. marinum*, or 2-hydroxy-*N*-phenylnaphthalene-1-carboxamide demonstrated MIC = 15.2 µmol/L against *M. kansasii*. The structure-activity relationships for all compounds are discussed.

## 1. Introduction

Since the 1980s morbidity due to bacterial infections has been rising again and today it has become an increasing worldwide threat. Mortality due to respiratory infections, AIDS and tuberculosis (TB) represents about 85% of all world mortality. The resistance of common pathogens to first-choice drugs increased up to 100% during the last decade, and resistance of some strains to second- or third-choice drugs is a great problem [1,2,3]. This is caused by general immunosuppression, a significant increase in the number of HIV-positive patients and development of resistance to commonly used drugs. The success of treatment is also decreased by development of cross-resistance or multidrug-resistant (MDR) strains. In general, suppressed immunity often leads to more lethal complications. For example, tuberculosis has re-emerged as a significant cause of global mortality. In 2011, there were an estimated 8.7 million new cases of TB (13% co-infected with HIV) and 1.4 million people died from TB, including almost one million deaths among HIV-negative individuals and 430,000 among people who were HIV-positive. The number of cases of MDR-TB notified in the 27 high MDR-TB burden countries is increasing and reached almost 60,000 worldwide in 2011. This is only one in five (19%) of the notified TB patients estimated to have MDR-TB [1,2]. Also, other non-tuberculous mycobacteria (NTM) are now recognized as significant human pathogens and cause difficult-to-treat or incurable diseases ending in deaths especially of immunocompromised patients [1,2,4,5,6]. The increasing number of infections and resistance of pathogens to drugs underline the importance of searching for new antimicrobial chemotherapeutics. The current situation even necessitates the re-engineering and repositioning of some old drug families to achieve effective control [4,6].

Salicylanilides (*N*-substituted hydroxybenzamides) represent compounds with a wide range of pharmacological activities, including anti-inflammatory [7], anthelmintic [8] and herbicidal [9,10,11] properties. Of these, at present antibacterial [9,10,11,12,13,14] and antimycobacterial [9,10,11,14,15] effects are becoming more and more important. The exact mechanisms of action are still under investigation, but these compounds are known to act as inhibitors of protein kinase epidermal growth factor receptor (EGFR PTK) [16]. Salicylanilides are generally designed to compete with ATP for binding in catalytic domain of tyrosine kinase [17,18] The latest studies specified them also as selective inhibitors of interleukin-12p40 production that plays a specific role in initiation, expansion, and control of cellular response to tuberculosis [19,20]. It was found that salicylanilides also inhibit bacterial enzymes, e.g., transglycosylases [21], d-alanine-d-alanine ligases [22] or sortase A [23].

The presence of an amide (-NHCO-) group is characteristic not only of various antibacterial drugs [7,24], but also of a number of herbicides acting as photosynthesis inhibitors [9,10,11,25,26,27], e.g., by reversibly binding to photosystem II [28,29]. As drugs and pesticides are designed to target particular biological systems, herbicides can also have molecular sites of action in mammals/non-plant organisms. Therefore many pharmaceutical companies have pesticide divisions and compounds generated by either division of the company are evaluated for both pesticide and pharmaceutical uses. In the past, some leading pesticides, (e.g., fluconazole), have become pharmaceuticals and *vice versa* [30,31,32]. Moreover, good correlation between microbiological activities and herbicidal or antialgal effects was found [10,11,27,33,34,35,36,37].

Investigation of synthesis and biological activity of ring-substituted 2-hydroxynaphthalene-1-carboxanilides is a follow-up contribution to understanding the structure-activity relationship within a series of modified salicylanilides. The design of these carboxanilides is based on the principle of ring analogy with salicylanilides. Thus, primary *in vitro* antibacterial and antimycobacterial screening of *N*-substituted 2-hydroxynaphthalene-1-carboxanilides as positional isomers of the *N*-substituted 3-hydroxynaphthalene-2-carboxanilides described by Kos *et al.* [11] was performed. A series of twenty-two compounds was also tested for their inhibitory effects on photosynthetic electron transport in spinach chloroplasts (*Spinacia* *oleracea* L.). The structure-activity relationships between the chemical structure, physical properties and *in vitro* biological activities of all the evaluated compounds are discussed.

## 2. Results and Discussion

### 2.1. Chemistry

**Scheme 1:**
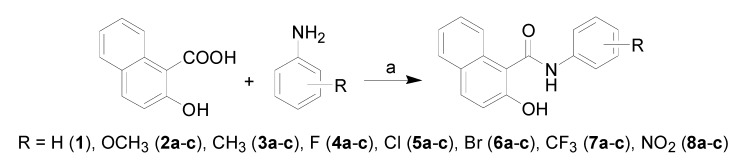
Synthesis of ring-substituted 2-hydroxynaphthalene-1-carboxanilides **1**–**8c**.

All the studied compounds were prepared according to Scheme 1. There are many methods for the preparation of *ortho*-hydroxycarboxanilides. The discussed compounds were synthesized by using modified microwave-assisted synthesis [11], thus synthesis of the target compounds was carried out in only one step with excellent yields. The condensation of 2-hydroxy-1-naphthoic acid with ring-substituted anilines using phosphorus trichloride in chlorobenzene under microwawe conditions yielded a series of twenty-two *N*-substituted 2-hydroxynaphthalene-1-carboxanilides **1**–**8c**.

*Reagents and conditions*: (a) PCl_3_, chlorobenzene, MW.

Within structure-activity relationship investigations various molecular descriptors (e.g., polar surface area, distributive parameters, electronic parameters, molar volume, number of rotatable bonds, and number of hydrogen bond donors or hydrogen bond acceptors, *etc.*), topological indexes or parameters describing physico-chemical properties (lipophilicity, solubility, ionizability, *etc.*) are used. Thus they represent numerical values that characterize properties of molecules that influence biological activity (receptor binding). In our previous studies biological activity was strongly influenced especially by lipophilicity and electronic parameters [9,10,11,14,25,26,27,38,39,40,41], expressed either as Hammett’s σ parameters or Taft polar substituent constants σ*.

**Figure 1:**
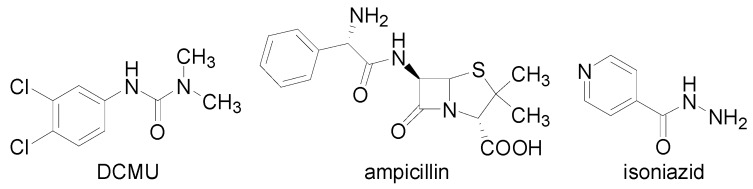
Chemical structures of 3-(3,4-dichlorophenyl)-1,1-dimethylurea (DCMU), ampicillin and isoniazid.

Lipophilicity as an important drug property has been studied and applied for decades, because drugs mostly cross biological membranes through passive transport, which strongly depends on their lipophilicity. Lipophilicity of the studied compounds **1**–**8c** and the associated relevant standards (Figure 1), was determined by RP-HPLC under isocratic conditions as capacity factor logarithm (log *k*) values. The results are shown in Table 1. For individual substituents in the anilide part of the discussed compounds also electronic Hammett’s σ parameters were predicted using the ACD/Percepta software; they ranged from −0.28/−0.27 (**2a**, R = 2-OCH_3_ / **2c**, R = 4-OCH_3_) to 0.71–0.78 (**9a**–**c**, R = 2-, 3-, 4-NO_2_).

2-Hydroxy-*N*-(4-methylphenyl)naphthalene-1-carboxamide (**3c**) showed the lowest lipophilicity while 2-hydroxy-*N*-phenylnaphthalene-1-carboxamide (**1**) had the highest, according to log *k* data. The whole series demonstrated significantly lower experimental lipophilicity of anilide substituted derivatives compared with the ring-substituted 3-hydroxynaphthalene-2-carboxanilides [11]. On the contrary, 2-hydroxy-*N*-phenylnaphthalene-1-carboxanilide (**1**) showed in comparison with 3-hydroxy-*N*-phenylnaphthalene-1-carboxanilide (log *k* = 0.6310) significantly higher experimentally determined lipophilicity (log *k* = 1.3016). Within the halogenated series the lipophilicity determined by log *k* values increases as follows: *meta* < *para* < *ortho*. Nevertheless the influence of R substituents on lipophilicity is as follows: CH_3_ < F < OCH_3_ < NO_2_ < Cl < Br < CF_3_ < H, in general.

### 2.2. Inhibition of Photosynthetic Electron Transport (PET) in Spinach Chloroplasts

**Figure 2:**
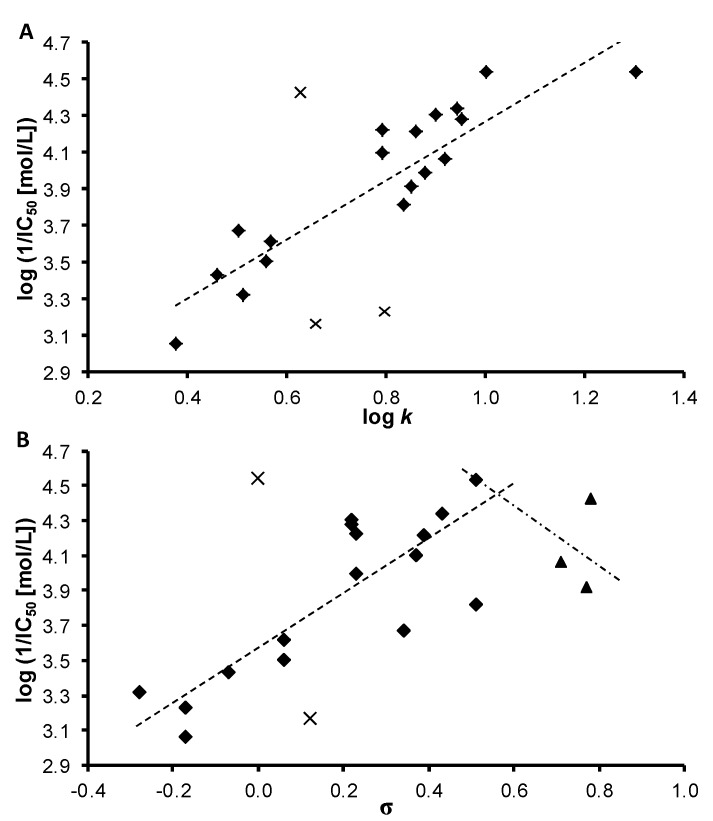
Relationships between PET inhibition log (1/IC_50_) [mol/L]) in spinach chloroplasts and lipophilicity expressed as log *k* (2**A**) or *N*-substituent electronic Hammett’s σ parameters (2**B**) of selected studied compounds (eliminated compounds are marked by crosses, nitro derivatives **8a**–**c** are marked by triangles).

The activity of the evaluated 2-hydroxynaphthalene-1-carboxanilides related to inhibition of photosynthetic electron transport (PET) in spinach (*Spinacia* *oleracea* L.) chloroplasts was medium or relative low with respect to the standard, see Table 1. IC_50_ value for compound **2c** (R = 4-OCH_3_) was not possible to determine due to its interaction with 2,6-dichlorophenol-indophenol (DCPIP). The most active PET inhibitors (IC_50_ = 29 µmol/L) were found to be compounds **1** (R = H) and **7c** (R = 4-CF_3_). The PET-inhibiting activity was expressed by negative logarithm of IC_50_ value (compound concentration in mol/L causing 50% inhibition of PET). Correlations between log(1/IC_50_ [mol/L]) and the lipophilicity of compounds expressed as log *k* or electronic properties of individual anilide substituents expressed as Hammett’s σ parameters were performed, see Figure 2.

**Table 1:** Structure of the discussed ring-substituted 2-hydroxynaphthalene-1-carboxanilides **1**–**8c**, experimentally determined values of lipophilicity log *k*, predicted electronic Hammett’s σ parameters, and IC_50_ values related to PET inhibition in spinach chloroplasts in comparison with 3-(3,4-dichlorophenyl)-1,1-dimethylurea (DCMU) standard, *in vitro* antibacterial activity (MIC) of compounds in comparison with ampicillin (APC) standard; *in vitro* antimycobacterial activity (MIC) of compounds in comparison with isoniazid (INH) standard, and *in vitro* cytotoxicity assay (LD_50_) of choice compounds.

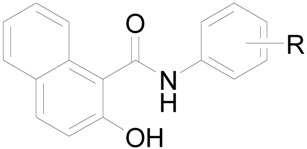													
Comp.	R	log *k*	σ*^a^*	[µmol/L]									
				PET IC_50_	MIC								LD_50_
					SA	MRSA 63718	MRSA 630	MRSA 3202	MM	MK	MS	MAP	
**1**	H	1.3016	0	**28.9**	>972	>972	243	122	**60.7**	**15.2**	486	950	>20
**2a**	2-OCH_3_	0.5121	−0.28	477	>873	>873	>873	>873	>873	>873	>873	852	–
**2b**	3-OCH_3_	0.6582	0.12	681	436	436	873	218	218	109	218	426	–
**2c**	4-OCH_3_	0.6342	−0.27	ND	>873	>873	>873	>873	109	218	436	852	–
**3a**	2-CH_3_	0.7962	−0.17	586	462	462	462	462	231	115	462	451	–
**3b**	3-CH_3_	0.4593	−0.07	372	231	462	231	231	231	115	231	451	–
**3c**	4-CH_3_	0.3751	−0.17	874	231	462	462	462	115	115	>923	451	–
**4a**	2-F	0.5664	0.06	243	228	455	455	455	228	114	228	203	–
**4b**	3-F	0.5025	0.34	213	228	>910	>910	228	>910	114	>910	>889	–
**4c**	4-F	0.5568	0.06	313	>910	>910	>910	>910	>910	>910	>910	>889	–
**5a**	2-Cl	0.8984	0.22	49.6	215	860	>860	>860	107	107	**107**	202	>20
**5b**	3-Cl	0.7904	0.37	79.7	>860	>860	>860	107	>860	53.7	215	840	–
**5c**	4-Cl	0.7908	0.23	59.2	215	>860	>860	107	>860	53.7	>860	420	–
**6a**	2-Br	0.9509	0.22	52.2	>748	>748	>748	>748	>748	93.5	>748	731	–
**6b**	3-Br	0.8595	0.39	61.1	>748	>748	>748	>748	>748	46.7	>748	731	–
**6c**	4-Br	0.8790	0.23	102	**47.0**	187	**47.0**	94.1	187	93.5	187	**175**	8.0
**7a**	2-CF_3_	0.8353	0.51	153	97.1	193	97.1	193	96.6	96.6	386	377	>20
**7b**	3-CF_3_	0.9411	0.43	45.6	>748	187	374	**94**	>748	93.5	>748	731	>20
**7c**	4-CF_3_	0.9994	0.51	**29.0**	>748	94	**94**	**47**	>748	**23.3**	>748	731	3.3
**8a**	2-NO_2_	0.8501	0.77	121	**26.0**	415	104	**52**	104	51.9	208	**195**	>20
**8b**	3-NO_2_	0.9187	0.71	86.4	208	**26.0**	208	208	**51.9**	104	208	405	>20
**8c**	4-NO_2_	0.6260	0.78	**37.5**	>830	830	415	104	**51.9**	415	208	811	2.5
**DCMU**	–	0.8801	0.6	1.9	–	–	–	–	–	–	–	–	–
**APC**	–	0.4337	–	–	5.7	>45.8	>45.8	>45.8	–	–	–	–	–
**INH**	–	0.0141	–	–	–	–	–	–	467	29.2	117	>1823	–

*^a^* calculated using ACD/Percepta ver. 2012 (Advanced Chemistry Development, Inc., Toronto, ON, Canada, 2012); *SA = Staphylococcus aureus* ATCC 29213, *MRSA =* clinical isolates of methicillin-resistant *Staphylococcus aureus* 63718, SA 630 and SA 3202 (National Institute of Public Health, Prague, Czech Republic); MM = *M. marinum* CAMP 5644, MK = *M. kansasii* DSM 44162, MM = *M. smegmatis* ATCC 700084 and clinical isolate MAP = *M. avium paratuberculosis* CIT03; ND = not determined due to its interaction with 2,6-dichlorophenol-indophenol (DCPIP).

The biological activity is affected by lipophilicity (see Table 1 and Figure 2A). In general, when the inactive anilide **2b** (3-OCH_3_) and **3a** (R = 2-CH_3_) as well as the compound **8c** (R = 4-NO_2_) were eliminated (marked by crosses), a linear dependence of log(1/IC_50_ [mol/L]) on log *k* for 19 *ortho*-, *meta*- and *para*-substituted derivatives could be observed. The correlation between log(1/IC_50_ [mol/L]) and log *k* could be expressed by the following equation:

log(1/IC_50_) = 2.613(±0.203) + 1.658(±0.251)log *k* (1)

r = 0.848, s = 0.244, F = 43.5, n = 19

On the other hand, PET inhibition is also influenced by the electronic properties of individual substituents, see Figure 2B. After elimination of unsubstituted compound **1** (IC_50_ = 28.9 µmol/L) and compound **2b** (R = 3-OCH_3_, IC_50_ = 681 µmol/L) for compounds with σ varying in the range from −0.28 to 0.51 a linear correlation between log(1/IC_50_ [mol/L]) on σ could be expressed by equation (2).


log(1/IC_50_) = 3.584 (± 0.085) + 1.493 (± 0.280)σ (2)

r = 0.818, s = 0.272, F = 28.3, n = 16

The linear correlation between log(1/IC_50_ [mol/L]) and log *k* for 16 compounds for which Equation (2) was evaluated is as follows:

log(1/IC_50_) = 2.449(±0.245) + 1.918(±0.323)log *k* (3)

r = 0.846, s = 0.252, F = 35.3, n = 16

For compounds with σ > 0.51, *i.e.*, **8a** (R = 2-NO_2_), **8b** (R = 3-NO_2_) and **8c** (R = 4-NO_2_) PET inhibiting activity decreased with increasing σ parameter. Bilinear dependence of PET inhibiting activity on electronic properties of R substituent was found for example with 2-benzylsulphanyl-benzimidazoles [42]. 

The use of both the abovementioned descriptors (log *k* and σ) in a multilinear correlation improved the statistical analysis results:

log(1/IC_50_) = 2.809(±0.234) + 1.223(±0.356)log *k* + 0.826(±0.286)σ (4)

r = 0.909, s = 0.204, F = 31.0, n = 16

The results of the statistical analyses above indicate that the biological activity of the tested compounds is significantly influenced by their lipophilicity and the σ parameter of the R substituent.

As the studied compounds were found to inhibit the Hill reaction, their site of action is situated in PS II. For more precise determination of their site of action diphenylcarbazide (DPC), an artificial electron donor acting in the intermediate Z^•^/D^•^ on the donor side of PS II [43] can be used. If addition of DPC to chloroplasts results in practically complete restoration of photosynthetic electron transport which was inhibited by one PET inhibitor, the inhibitory site of action of this inhibitor is situated exclusively on the donor side of PS II, in the section between oxygen evolving complex and Z^•^/D^•^ intermediate. On the other hand, if the site of action of certain PS II inhibitor would be situated on the acceptor side of PS II, in the section between PS II reaction centre (P680) and secondary quinone acceptor Q_B_, the supply of electrons by DPC acting in Z^•^/D^•^ intermediate would not be able to secure complete PET restoration. The results of DPC experiment confirmed that the site of action of the studied compounds is situated only on the donor side of PS II. Similar site of action in the photosynthetic electron transport chain was found for substituted salicylanilides [38], *N*-substituted 2-aminobenzothiazoles [27], *N*-phenylcarbamates [39], and 4-chloro-2-(chlorophenylcarbamoyl)phenyl alkylcarbamates [40].

**Figure 3:**
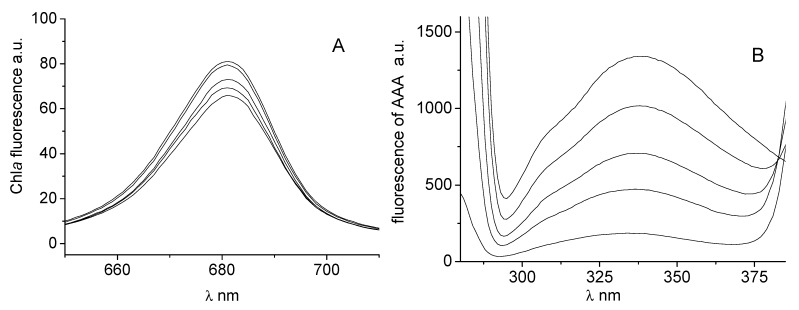
Emission fluorescence spectra of chlorophyll *a* in suspension of spinach chloroplasts without and with compound **1** (c = 0, 0.059, 0.117, 0.234 and 0.468 mmol/L; curves from top to bottom) (**A**), and emission fluorescence spectra of aromatic amino acids in suspension of spinach chloroplasts without and with **1** (c = 0, 0.012, 0.029, 0.059, 0.117 mmol/L) (**B**).

The effects of studied compounds on the photosynthetic centres of chloroplasts were investigated by studying chlorophyll *a* fluorescence. Figure 3A shows the effect of compound **1** on the chlorophyll *a* fluorescence emission band at 686 nm. The decreased intensity of this emission band belonging to the chlorophyll-protein complexes mainly in photosystem (PS) II [44] suggested PS II as the site of action of the studied inhibitor.

Binding of PET inhibitors to aromatic amino acids (AAA) occurring in photosynthetic proteins of spinach chloroplasts situated in PS II contributes to PET inhibition. The interaction of AAA with PET inhibitors can be studied by the quenching of AAA fluorescence at 334 nm Figure 3B presents fluorescence emission spectra of aromatic amino acids of untreated spinach chloroplasts and of chloroplasts treated with increasing concentrations of compound **1** (see Figure 3B). Increasing concentration of **1** resulted in strong decline of AAA fluorescence and similar effects exhibited also further studied compounds. Decrease of Chl*a* and AAA fluorescence in spinach chloroplasts was observed previously in the presence of substituted *N-*benzylpyrazine-2-carboxamides [41] and *N*-substituted 2-aminobenzothiazoles [27].

### 2.3. In Vitro Antibacterial Susceptibility Testing

The discussed compounds were tested for their *in vitro* antibacterial activity against *Staphylococcus aureus* ATCC 29213 (SA) and three methicillin-resistant *S. aureus* (MRSA 63718, SA 630, SA 3202), see Table 1. *S. aureus* and especially MRSA strains were selected, because they caused life-threatening nosocomial infections for decades and have recently become a significant threat for community acquired infections and livestock associated infections with high levels of morbidity and mortality. Methicillin resistance is connected with not only clinically inadequate susceptibility to all β-lactam antibiotics but usually to other antimicrobial drugs as well [45,46]. Therefore there is an urgent need to develop new, potent, and fast acting anti-tuberculosis and anti-MRSA drugs.

Although salicylanilides seem to be promising antibacterial agent candidates [13], most of the compounds showed only moderate activity, except for 2-hydroxy-*N*-(2-nitrophenyl)naphthalene-1-carboxamide (**8a**), 2-hydroxy-*N*-(3-nitrophenyl)naphthalene-1-carboxamide (**8b**), 2-hydroxy-*N*-(4-bromophenyl)naphthalene-1-carboxamide (**6c**) and 2-hydroxy-*N*-(4-trifluoromethylphenyl)-naphthalene-1-carboxamide (**7c**), whose activity, especially against MRSA, was comparable with or higher than that of the standard, see Table 1. Nevertheless due to the moderate activity of the rest of the compounds, no thorough structure-activity relationships could be established. In general, it can be stated that antibacterial activity is positively influenced by higher lipophilicity of compounds with simultaneous substitution of the anilide ring by electron-withdrawing moieties.

In our previous study of 3-hydroxynaphthalene-2-carboxanilides [11] only 3-hydroxy-*N*-(2-methoxyphenyl)naphthalene-2-carboxamide showed biological activity (MIC = 55.0 µmol/L) against *S. aureus*, as well as all the MRSA strains. The lipophilicity of this compound is log *k* = 0.69, and 2-methoxy moiety is a significant electron donor (σ = −0.28). Inactivity of the rest of those derivatives may be caused by their relatively low solubility in aqueous media and different properties of compounds affecting their biological activity. Similarly, as differences in lipo/hydrophilic properties of 3-hydroxynaphthalene-2-carboxanilides *vs.* 2-hydroxynaphthalene-1-carboxanilides were mentioned above, different substituents in the anilide ring were found as being favourable in other series of hydroxyarylcarboxanilides [9,10,14,25].

### 2.4. *In Vitro* Antimycobacterial Evaluation

The evaluation of the *in vitro* antimycobacterial activity of the compounds was performed against *Mycobacterium marinum* CAMP 5644 (MM), *M. kansasii* DSM 44162 (MK), *M. smegmatis* ATCC 700084 (MS) and a clinical isolate *M. avium paratuberculosis* CIT03 (MAP), see Table 1. The genus *Mycobacterium* consists of a closely related group of fast and slow-growing species. *M. tuberculosis* causes one of the most serious human infections, (tuberculosis). Difficulties should be considered while studying *M. tuberculosis*—especially a slow growth rate and the requirement of working in high containment biosafety facilities. To lower risk and make manipulation in the laboratory easier, surrogate model pathogens for *M. tuberculosis* can be used in laboratory studies. *M.* *smegmatis* is an ideal representative of a fast-growing nonpathogenic microorganism particularly useful in studying basic cellular processes of special relevance to pathogenic mycobacteria. *M. marinum* is very closely related to the *M. tuberculosis* and it is the cause of TB-like infections in poikilothermic organisms, especially frogs and fish. *M. marinum* is a good model for studying especially because of the lower risk for laboratory workers, genetic relatedness and similar pathology to human TB [47,48,49]. However, because of *M. tuberculosis*, the pathogenic role of NTM in humans was overshadowed for a long time. *M. kansasii*, the most virulent of the NTM, causes non-tuberculous mycobacterial lung infections which are very common nowadays and can be indistinguishable from tuberculosis [50]. *M. avium paratuberculosis* is suspected to be a causative agent in gastrointestinal diseases, is resistant to standard antimycobacterial therapy, but is susceptible to some standard antibiotics. However, the resistance to these antibiotics develop fast [51].

As shown in Table 1, isoniazid was active only against *M. kansasii*, whereas the target compounds showed comparable with or higher effectiveness than that of the isoniazid. Although compounds showed a wide range of activity that did not reach a level equivalent to “classical antituberculotics”, some general structure-activity relationships could be established.

Compounds **8b** (R = 3-NO_2_) and **8c** (R = 4-NO_2_) demonstrated about 9-fold higher activity (MIC ca. 52 µmol/L) against *M.* *marinum* than isoniazid, unsubstituted compound **1** and **7c** (R = 4-CF_3_) showed higher effect against *M.* *kansasii* than isoniazid as well as **5a** (R = 2-Cl) against *M.* *smegmatis*. Almost all the compounds expressed higher activity against *M. avium paratuberculosis* than isoniazid, nevertheless **6c** (R = 4-Br) was the most active. Based on these facts and data presented in Table 1 it can be concluded that activity decreased with change of anilide substituent position as follows: *para* > *meta* > *ortho*. Further parameters influencing activity can involve lipophilicity and electronic properties of the individual substituents. In general it can be stated that the effects against all four mycobacterial strains increased with higher lipophilicity and also electron-withdrawing properties of individual substituents are more advantageous than electron donor substituents.

Generally it can be stated that from the tested compounds *para*- and *meta*-substituted derivatives or non-substituted compound **1** expressed higher antimycobacterial activity than *ortho*-substituted ones. This fact is probably caused by a steric effect of a spatially-close moiety. The proximity of the *ortho*-substituent to the carboxamide group on the aniline ring led to the twist of the aniline ring plain towards the carboxamide group, *i.e.*, to the whole naphthalene core, while *meta*- and especially *para*-substitution lead to planar structure that, when the substituent is more lipophilic, easier permeates through various types of membranes [52]. Thus, the lipophilicity of *meta*- and *para*-substituted derivatives was found as the factor conducive to the activity of such structures. As compounds with electron-withdrawing substituents expressed antimycobacterial activities, it can be stated that these components affecting electronic density (charge) at the carbonyl moiety influence the potential of binding of the carboxamide group to possible binding sites in a mycobacterial cell [52]. Janin suggests establishing the hypothesis that carboxamides can interfere with the mycobacterial proton pump F_0_F_1_H^+^ATPase or they can inhibit biosynthesis of amino acid [24]. Kratky *et al.* recently reported the ability of salicylanilide derivatives to inhibit two essential mycobacterial enzymes; methionine aminopeptidase (performs an important function in different growth phases) and isocitrate lyase (essential for the metabolism of fatty acids). Both are necessary for the maintenance of the latent tuberculosis infection [16].

Previously presented 3-hydroxynaphthalene-2-carboxanilides [11] with different substituents showed antimycobacterial activity. As preferred substitutions with activity against *M. marinum* and *M. kansasii* 2-fluoro, 2-methoxy and 3-methyl moieties were found. In general it can be stated that in both series, derivatives with lipophilicity log *k* > 0.62 can show antimycobacterial effect. Within 3-hydroxynaphthalene-2-carboxanilides rather electron-neutral or slightly electron-withdrawing substituents are preferable [11], while in the series of 2-hydroxynaphthalene-1-carboxanilides substituents with significant electron-withdrawing effect are favoured.

### 2.5. *In Vitro* Cytotoxicity Assay

The *in vitro* primary screening of cytotoxicity of the selected compounds was performed using the human monocytic leukemia THP-1 cell line. The cytotoxicity was evaluated as the LD_50_ value (LD_50_–lethal dose to 50% of the cell population), see Table 1. Compounds were not soluble above concentrations 20 μmol/L, the highest concentration prepared in DMSO for the toxicity assay was 20.0 μmol/L. Treatment with 20 μmol/L of **1**, **5a**, **7a**, **7b**, **8a** and **8b** did not lead to significant lethal effects on THP-1 cells. The strongest effect on cell viability was detected in compounds **6c** (LD_50_ = 8.0 µmol/L), **7c** (LD_50_ = 3.3 µmol/L), and **8c** (LD_50_ = 2.5 µmol/L); however, their toxicity is still not comparable with those of camptothecin (0.16 ± 0.07 µmol/L) assessed in this line formerly. It is evident that cytotoxicity within the series of carboxanilides is related only to *para*-position of substituents and increases with electron-withdrawing properties of substituents, see Table 1. Based on these observations it is important to conclude that unsubstituted **1** (R = H), **8a** (R = 2-NO_2_) and **8b** (R = 3-NO_2_) or **5a** (R = 2-Cl) can be considered as non-toxic agents for subsequent design of novel antimicrobial agents.

## 3. Experimental

### 3.1. General

All reagents were purchased from Aldrich (Sigma-Aldrich, St. Louis, MO, USA) and Merck (Darmstadt, Germany). Reactions were carried out in StartSYNTH microwave labstation (Milestone, Italy). The melting points were determined on Kofler hot-plate apparatus (HMK Franz Kustner, Germany) and are uncorrected. Infrared (IR) spectra were recorded on a Smart MIRacle™ ATR ZnSe for Nicolet™ Impact 400 FT-IR spectrometer (Thermo Electron Corporation, West Palm Beach, FL, USA). The spectra were obtained by accumulation of 256 scans with 2 cm^−1^ resolution in the region of 4000–600 cm^−1^. All ^1^H and ^13^C-NMR spectra were recorded on a Bruker Avance III 400 MHz FT-NMR spectrometer (400 MHz for ^1^H and 100 MHz for ^13^C, Bruker Comp., Karlsruhe, Germany). Chemical shifts are reported in ppm (δ) using signal of the solvent (DMSO-*d_6_*) as the reference (2.500, resp. 39.50). Mass spectra were measured using a LTQ Orbitrap Hybrid Mass Spectrometer (Thermo Electron Corporation) with direct injection into an APCI source (400 °C) in the positive mode.

### 3.2. Synthesis

#### General Procedure for Synthesis of Carboxamide Derivatives **1**–**8c**

2-Hydroxynaphthalene-1-carboxylic acid (5.3 mmol) and the corresponding substituted aniline (5.3 mmol) were suspended in dry chlorobenzene (30 mL). Phosphorous trichloride (2.65 mmol) was added dropwise, and the reacting mixture was heated in the microwave reactor at maximal allowed power 500 W and 130 °C, using infrared flask-surface control of temperature, for 15 min. The solvent was evaporated under reduced pressure, the solid residue washed with 2M HCl, and the crude product was recrystallized from aqueous ethanol. Studied compounds **1**–**8c** are presented in Table 1.

*2-Hydroxy-N-phenylnaphthalene-1-carboxamide* (**1**). Yield 80%; Mp 172–173 °C (171–172 °C [53], 182 °C [54]); IR (cm^−1^): 3180, 3050, 1637, 1627, 1597, 1536, 1513, 1497, 1442, 1431, 1328, 1306, 1234, 1203, 963, 813, 740, 693; ^1^H-NMR (DMSO-*d_6_*), δ: 11.11 (s, 1H), 8.20 (d, 1H, *J* = 8.5 Hz), 7.97 (br. s, 1H), 7.86 (t, 2H, *J* = 8.2 Hz), 7.62 (d, 2H, *J* = 7.8 Hz), 7.48 (ddd, 1H, *J* = 8.0 Hz, *J* = 7.0 Hz, *J* = 1.3 Hz), 7.44–7.36 (m, 3H), 7.25–7.18 (m, 2H); ^13^C-NMR (DMSO-*d_6_*), δ: 168.46, 159.81, 136.90, 134.51, 130.46, 129.73, 129.32, 128.89, 128.63, 125.30, 123.72, 122.29, 120.74, 119.36, 109.91; HR-MS: for C_17_H_13_NO_2_ [M+H]^+^ calculated 264.1019 *m*/*z*, found 264.1023 *m*/*z*.

*2-Hydroxy-N-(2-methoxyphenyl)naphthalene-1-carboxamide* (**2a**). Yield 76%; Mp 169–170 °C; IR (cm^−1^): 3283, 1645, 1600, 1528, 1513,1478, 1459, 1431, 1337, 1281, 1245, 1113, 1022, 966, 894, 818, 743, 735; ^1^H-NMR (DMSO-*d_6_*), δ: 10.42 (br. s, 1H), 9.34 (s, 1H), 8.28 (dd, 1H, *J* = 7.8 Hz, *J* = 1.0 Hz), 8.00 (d, 1H, *J* = 8.5 Hz), 7.87 (d, 1H, *J* = 9.0 Hz), 7.84 (d, 1H, *J* = 8.3 Hz), 7.48 (ddd, 1H, *J* = 8.0 Hz, *J* = 7.0 Hz, *J* = 1.3 Hz), 7.33 (ddd, 1H, *J* = 8.0 Hz, *J* = 7.0 Hz, *J* = 1.3 Hz), 7.25 (d, 1H, *J* = 8.8 Hz), 7.16–7.07 (m, 2H), 7.01 (dt, 1H, *J* = 7.5 Hz, *J* = 1.0 Hz), 3.82 (s, 3H); ^13^C-NMR (DMSO-*d_6_*), δ: 165.31, 152.19, 149.45, 131.76, 130.90, 128.02, 127.68, 127.64, 127.00, 124.47, 123.95, 123.08, 121.37, 120.45, 118.30, 117.13, 111.25, 55.82; HR-MS: for C_18_H_15_NO_3_ [M+H]^+^ calculated 294.1125 *m*/*z*, found 294.1133 *m*/*z*.

*2-Hydroxy-N-(3-methoxyphenyl)naphthalene-1-carboxamide* (**2b**). Yield 77%; Mp 147–149 °C; IR (cm^−1^): 3115, 1642, 1607, 1538, 1512, 1447, 1329, 1240, 1202, 1157, 1029, 815, 776, 755, 738, 684; ^1^H-NMR (DMSO-*d_6_*), δ: 10.37 (s, 1H), 10.12 (br. s, 1H), 7.87–7.84 (m, 2H), 7.68 (d, 1H, *J* = 8.3 Hz), 7.55 (s, 1H), 7.46 (ddd, 1H, *J* = 8.0 Hz, *J* = 7.0 Hz, *J* = 1.3 Hz), 7.36–7.31 (m, 2H), 7.26–7.23 (m, 2H), 6.68 (d, 1H, *J* = 7.8 Hz), 3.76 (s, 3H); ^13^C-NMR (DMSO-*d_6_*), δ: 165.79, 159.54, 151.61, 140.84, 131.40, 130.13, 129.47, 127.97, 127.38, 126.97, 123.41, 123.00, 118.62, 118.35, 111.60, 108.71, 105.07, 54.99; HR-MS: for C_18_H_15_NO_3_ [M+H]^+^ calculated 294.1125 *m*/*z*, found 294.1132 *m*/*z*.

*2-Hydroxy-N-(4-methoxyphenyl)naphthalene-1-carboxamide* (**2c**). Yield 74%; Mp 168-170°C; IR (cm^−1^): 3262, 1625, 1538, 1506, 1462, 1413, 1247, 1232, 1204, 1178, 1027, 828, 792, 712; ^1^H-NMR (DMSO-*d_6_*), δ: 10.24 (s, 1H), 10.08 (br. s, 1H), 7.84 (d, 2H, *J* = 8.8 Hz), 7.75–7.72 (m, 2H), 7.69 (d, 2H, *J* = 8.5 Hz), 7.46 (ddd, 1H, *J* = 8.0 Hz, *J* = 7.0, *J* = 1.3 Hz), 7.32 (ddd, 1H, *J* = 8.0 Hz, *J* = 7.0 Hz, *J* = 1.3 Hz), 7.25 (d, 1H, *J* = 8.8 Hz), 6.94 (d, 1H, *J* = 8.8 Hz), 3.75 (s, 3H); ^13^C-NMR (DMSO-*d_6_*), δ: 165.17, 155.24, 151.56, 132.93, 131.50, 130.00, 127.95, 127.41, 126.90, 123.52, 122.96, 120.72, 118.76, 118.38, 113.81, 55.21; HR-MS: for C_18_H_15_NO_3_ [M+H]^+^ calculated 294.1125 *m*/*z*, found 294.1133 *m*/*z*.

*2-Hydroxy-N-(2-methylphenyl)naphthalene-1-carboxamide* (**3a**). Yield 69%; Mp 127–130 °C; IR (cm^−1^): 3176, 1615, 1599, 1574, 1519, 1456, 1433, 1315, 1276, 1252, 1212, 1148, 1040, 969, 900, 818, 752, 743; ^1^H-NMR (DMSO-*d_6_*), δ: 10.16 (br. s, 1H), 9.80 (s, 1H), 7.88–7.84 (m, 3H), 7.60 (d, 1H, *J* = 7.8 Hz), 7.50 (ddd, 1H, *J* = 8.0 Hz, *J* = 7.0 Hz, *J* = 1.3 Hz), 7.34 (ddd, 1H, *J* = 8.0 Hz, *J* = 7.0 Hz, *J* = 1.3 Hz), 7.28–7.23 (m, 3H), 7.15 (t, 1H, *J* = 7.3 Hz) 2.35 (s, 3H); ^13^C-NMR (DMSO-*d_6_*), δ: 165.64, 151.79, 136.45, 132.54, 131.67, 131.31, 131.30, 130.09, 127.97, 127.49, 126.89, 125.88, 125.86, 125.41, 123.64, 122.96, 118.36, 18.08; HR-MS: for C_18_H_15_NO_2_ [M+H]^+^ calculated 278.1176 *m*/*z*, found 278.1183 *m*/*z*.

*2-Hydroxy-N-(3-methylphenyl)naphthalene-1-carboxamide* (**3b**). Yield 74%; Mp 145–148 °C; IR (cm^−1^): 3343, 3206, 3029, 1628, 1614, 1601,1575, 1527, 1435, 1274, 1229, 1211, 1146, 1042, 967, 819, 773, 745, 687; ^1^H-NMR (DMSO-*d_6_*), δ: 10.32 (s, 1H), 10.10 (br. s, 1H), 7.87–7.84 (m, 2H), 7.71 (s, 1H), 7.69 (d, 1H, *J* = 8.5 Hz), 7.57 (d, 1H, *J* = 8.0 Hz), 7.46 (ddd, 1H, *J* = 8.0 Hz, *J* = 7.0 Hz, *J* = 1.3 Hz), 7.33 (ddd, 1H, *J* = 8.0 Hz, *J* = 7.0 Hz, *J* = 1.3 Hz), 7.26 (d, 1H, *J* = 8.8 Hz), 8.23 (t, 1H, *J* = 7.5 Hz), 6.92 (d, 1H, *J* = 7.5 Hz), 2.32 (s, 3H); ^13^C-NMR (DMSO-*d_6_*), δ: 165.69, 151.59, 139.60, 137.84, 131.45, 130.06, 128.52, 127.97, 127.40, 126.94, 123.98, 123.44, 122.99, 119.81, 118.72, 118.38, 116.53, 21.32; HR-MS: for C_18_H_15_NO_2_ [M+H]^+^ calculated 278.1176 *m*/*z*, found 278.1182 *m*/*z*.

*2-Hydroxy-N-(4-methylphenyl)naphthalene-1-carboxamide* (**3c**). Yield 77%; Mp 143–145 °C; IR (cm^−1^): 3291, 1637, 1599, 1537, 1513, 1435, 1323, 1299, 1252, 1210, 1044, 963, 892, 806, 739; ^1^H-NMR (DMSO-*d_6_*), δ: 10.30 (s, 1H), 10.09 (br. s, 1H), 7.86-7.83 (m, 2H), 7.72–7.68 (m, 3H), 7.46 (ddd, 1H, *J* = 8.0 Hz, *J* = 7.0 Hz, *J* = 1.3 Hz), 7.32 (ddd, 1H, *J* = 8.0 Hz, *J* = 7.0 Hz, *J* = 1.3 Hz), 7.25 (d, 1H, *J* = 9.0 Hz), 7.16 (d, 1H, *J* = 8.3 Hz), 2.29 (s, 3H); ^13^C-NMR (DMSO-*d_6_*), δ: 165.49, 151.58, 137.23, 132.15, 131.48, 130.04, 129.06, 127.97, 127.41, 126.93, 126.92, 123.47, 122.98, 119.27, 118.38, 20.54; HR-MS: for C_18_H_15_NO_2_ [M+H]^+^ calculated 278.1176 *m*/*z*, found 278.1180 *m*/*z*.

*N-(2-fluorophenyl)-2-hydroxynaphthalene-1-carboxamide* (**4a**). Yield 64%; Mp 150–152 °C; IR (cm^−1^): 3256, 1622, 1605, 1580, 1524, 1502, 1460, 1403, 1327, 1266, 1220, 1201, 1148, 1105, 970, 861, 819, 795, 763, 753, 704; ^1^H-NMR (DMSO-*d_6_*), δ: 10.22 (br. s, 1H), 10.17 (s, 1H), 7.99–7.95 (m, 2H), 7.88-7.83 (m, 3H), 7.49 (ddd, 1H, *J* = 8.0 Hz, *J* = 7.0 Hz, *J* = 1.3 Hz), 7.34 (ddd, 1H, *J* = 8.0 Hz, *J* = 7.0 Hz, *J* = 1.3 Hz), 7.33–7.27 (m, 1H), 7.26–7.22 (m, 3H); ^13^C-NMR (DMSO-*d_6_*), δ: 156.92, 154.48 (d, *J* = 246.5 Hz), 151.95, 131.60, 130.41, 127.99, 127.47, 126.96, 126.13 (d, *J* = 12.5 Hz), 125.88 (d, *J* = 8.1 Hz), 125.35 (d, *J* = 1.5 Hz), 124.32 (d, *J* = 3.7 Hz), 123.56, 122.99, 118.33, 117.72, 115.68 (d, *J* = 19.1 Hz); HR-MS: for C_17_H_12_NO_2_F [M+H]^+^ calculated 282.0925 *m*/*z*, found 282.0931 *m*/*z*.

*N-(3-fluorophenyl)-2-hydroxynaphthalene-1-carboxamide* (**4b**). Yield 68%; Mp 187–190 °C; IR (cm^−1^): 3371, 3284, 1640, 1611, 1586, 1514, 1445, 1430, 1279, 1238, 1212, 1145, 1039, 973, 956, 807, 766, 676; ^1^H-NMR (DMSO-*d_6_*), δ: 10.62 (s, 1H), 10.18 (br. s, 1H), 7.89–7.81 (m, 3H), 7.67 (d, 1H, *J* = 8.5 Hz), 7.53–7.51 (m, 1H), 7.47 (ddd, 1H, *J* = 8.0 Hz, *J* = 7.0 Hz, *J* = 1.3 Hz), 7.39 (ddd, 1H, *J* = 8.0 Hz, *J* = 7.0 Hz, *J* = 1.3 Hz), 7.38–7.31 (m, 1H), 7.26 (d, 1H, *J* = 9.0 Hz), 6.93 (ddd, 1H, *J* = 8.7 Hz, *J* = 2.8 Hz, *J* = 0.7 Hz); ^13^C-NMR (DMSO-*d_6_*), δ: 166.10, 162.20 (d, *J* = 241.4 Hz), 151.73, 141.33 (d, *J* = 11.0 Hz), 131.32, 130.37, 130.37 (d, *J* = 9.5 Hz), 128.02, 127.37, 127.10, 123.31, 123.09, 118.33, 118.23, 115.03 (d, *J* = 2.9 Hz), 109.75 (d, *J* = 21.3 Hz), 105.99 (d, *J* = 26.4 Hz); HR-MS: for C_17_H_12_NO_2_F [M+H]^+^ calculated 282.0925 *m*/*z*, found 282.0933 *m*/*z*.

*N-(4-fluorophenyl)-2-hydroxynaphthalene-1-carboxamide* (**4c**). Yield 72%; Mp 210–212 °C; IR (cm^−1^): 3266, 1638, 1615, 1547, 1507, 1434, 1404, 1211, 1147, 1098, 1040, 961, 827, 810, 739; ^1^H-NMR (DMSO-*d_6_*), δ: 10.46 (s, 1H), 10.13 (br. s, 1H), 7.87-7.82 (m, 4H), 7.68 (d, 1H, *J* = 8.3 Hz), 7.46 (ddd, 1H, *J* = 8.0 Hz, *J* = 7.0 Hz, *J* = 1.3 Hz), 7.33 (ddd, 1H, *J* = 8.0 Hz, *J* = 7.0 Hz, *J* = 1.3 Hz), 7.26 (d, 1H, *J* = 9.0 Hz), 7.23–7.18 (m, 2H); ^13^C-NMR (DMSO-*d_6_*), δ: 165.60, 158.04 (d, *J* = 239.2 Hz), 151.65, 136.08 (d, *J* = 2.9 Hz), 131.41, 130.20, 127.99, 127.40, 127.02, 123.41, 123.04, 120.94 (d, *J* = 8.1 Hz), 118.46, 118.36, 115.27 (d, *J* = 22.0 Hz); HR-MS: for C_17_H_12_NO_2_F [M+H]^+^ calculated 282.0925 *m*/*z*, found 282.0930 *m*/*z*.

*N-(2-chlorophenyl)-2-hydroxynaphthalene-1-carboxamide* (**5a**). Yield 62%; Mp 138–139 °C; IR (cm^−1^): 3271, 1625, 1614, 1580, 1518, 1431, 1402, 1321, 1283, 1229, 1196, 1156, 1032, 821, 793, 762, 689; ^1^H-NMR (DMSO-*d_6_*), δ: 10.38 (br. s, 1H), 9.99 (s, 1H), 8.00 (d, 1H, *J* = 8.3 Hz), 7.97 (d, 1H, *J* = 7.8 Hz), 7.88 (d, 1H, *J* = 9.0 Hz), 7.85 (d, 1H, *J* = 8.5 Hz), 7.55 (d, 1H, *J* = 8.0 Hz), 7.50 (ddd, 1H, *J* = 8.0 Hz, *J* = 7.0 Hz, *J* = 1.3 Hz), 7.42 (t, 1H, *J* = 7.5 Hz), 7.34 (ddd, 1H, *J* = 8.0 Hz, *J* = 7.0 Hz, *J* = 1.3 Hz), 7.28–7.24 (m, 2H); ^13^C-NMR (DMSO-*d_6_*), δ: 165.81, 152.29, 135.15, 131.70, 130.80, 129.56, 128.03, 127.57, 127.50, 127.02, 126.46, 126.41, 126.40, 123.81, 123.07, 118.30, 117.09; HR-MS: for C_17_H_12_NO_2_Cl [M+H]^+^ calculated 298.0629 *m*/*z*, found 298.0636 *m*/*z*.

*N-(3-chlorophenyl)-2-hydroxynaphthalene-1-carboxamide* (**5b**). Yield 64%; Mp 191–194 °C; IR (cm^−1^): 3358, 3297, 1640, 1589, 1538, 1514, 1474, 1422, 1353, 1312, 1265, 1212, 1145, 1038, 963, 911, 808, 770, 746, 676; ^1^H-NMR (DMSO-*d_6_*), δ: 7.61 (s, 1H), 10.19 (br. s, 1H), 8.06 (t, 1H, *J* = 1.9 Hz), 7.89–7.85 (m, 2H), 7.69–7.64 (m, 2H), 7.47 (ddd, 1H, *J* = 8.0 Hz, *J* = 7.0 Hz, *J* = 1.3 Hz), 7.38 (t, 1H, *J* = 8.2 Hz), 7.33 (ddd, 1H, *J* = 8.0 Hz, *J* = 7.0 Hz, *J* = 1.3 Hz), 7.26 (d, 1H, *J* = 9.0 Hz), 7.16 (ddd, 1H, *J* = 8.0 Hz, *J* = 2.0 Hz, *J* = 1.0 Hz); ^13^C-NMR (DMSO-*d_6_*), δ: 166.13, 151.75, 141.05, 133.11, 131.32, 130.46, 130.41, 128.03, 127.38, 127.13, 123.31, 123.11, 123.03, 118.70, 118.34, 118.18, 117.66; HR-MS: for C_17_H_12_NO_2_Cl [M+H]^+^ calculated 298.0629 *m*/*z*, found 298.0636 *m*/*z*.

*N-(4-chlorophenyl)-2-hydroxynaphthalene-1-carboxamide* (**5c**). Yield 61%; Mp 193–195 °C (193 °C [55]); IR (cm^−1^): 3293, 1639, 1597, 1538, 1514, 1492, 1435, 1397, 1321, 1300, 1269, 1250, 1211, 1092, 810; ^1^H-NMR (DMSO-*d_6_*), δ: 10.55 (s, 1H), 10.17 (br. s, 1H), 7.88–7.84 (m, 4H), 7.67 (d, 1H, *J* = 8.5 Hz), 7.46 (ddd, 1H, *J* = 8.0 Hz, *J* = 7.0 Hz, *J* = 1.3 Hz), 7.42 (d, 2H, *J* = 9.0 Hz), 7.33 (ddd, 1H, *J* = 8.0 Hz, *J* = 7.0 Hz, *J* = 1.3 Hz), 7.26 (d, 1H, *J* = 8.8 Hz); ^13^C-NMR (DMSO-*d_6_*), δ: 165.88, 151.70, 138.60, 131.36, 130.31, 128.64, 128.02, 127.39, 127.08, 126.83, 123.35, 123.07, 120.79, 118.36, 118.33; HR-MS: for C_17_H_12_NO_2_Cl [M+H]^+^ calculated 298.0629 *m*/*z*, found 298.0635 *m*/*z*.

*N-(2-bromophenyl)-2-hydroxynaphthalene-1-carboxamide* (**6a**). Yield 74%; Mp 158–160 °C; IR (cm^−1^): 3267, 1625, 1615, 1575, 1516, 1460, 1425, 1401, 1319, 1281, 1221, 1195, 1155, 1141, 1022, 821, 762; ^1^H-NMR (DMSO-*d_6_*), δ: 10.40 (br. s, 1H), 9.90 (s, 1H), 8.04 (d, 1H, *J* = 8.3 Hz), 7.93 (d, 1H, *J* = 7.8 Hz), 7.88 (d, 1H, *J* = 9.0 Hz), 7.85 (d, 1H, *J* = 8.3 Hz), 7.72 (d, 1H, *J* = 7.8 Hz), 7.52–7.45 (m, 2H), 7.34 (ddd, 1H, *J* = 8.0 Hz, *J* = 7.0 Hz, *J* = 1.3 Hz), 7.26 (d, 1H, *J* = 8.8 Hz), 7.19 (d, 1H, *J* = 7.4 Hz); ^13^C-NMR (DMSO-*d_6_*), δ: 165.81, 152.33, 136.54, 132.75, 131.68, 130.83, 128.12, 128.02, 127.58, 127.01, 126.99, 126.84, 123.91, 123.08, 118.29, 117.82, 117.04; HR-MS: for C_17_H_12_NO_2_Br [M+H]^+^ calculated 342.0124 *m*/*z*, found 342.0133 *m*/*z*.

*N-(3-bromophenyl)-2-hydroxynaphthalene-1-carboxamide* (**6b**). Yield 72%; Mp 195–198 °C; IR (cm^−1^): 3354, 3302, 1640, 1586, 1532, 15313, 1471, 1436, 1418, 1353, 1317, 1271, 1264, 1252, 1212, 963, 901, 857, 808, 766, 745, 674; ^1^H-NMR (DMSO-*d_6_*), δ: 10.59 (s, 1H), 10.19 (br. s, 1H), 8.20 (s, 1H), 7.88–7.84 (m, 2H), 7.69–7.66 (m, 2H), 7.47 (ddd, 1H, *J* = 8.0 Hz, *J* = 7.0 Hz, *J* = 1.3 Hz), 7.35–7.24 (m, 4H); ^13^C-NMR (DMSO-*d_6_*), δ: 166.10, 151.73, 141.18, 131.30, 130.76, 130.39, 128.02, 127.36, 127.12, 125.92, 123.30, 123.09, 121.61, 121.54, 118.33, 118.15, 118.03; HR-MS: for C_17_H_12_NO_2_Br [M+H]^+^ calculated 342.0124 *m*/*z*, found 342.0132 *m*/*z*.

*N-(4-bromophenyl)-2-hydroxynaphthalene-1-carboxamide* (**6c**). Yield 68%; Mp 188–190 °C; IR (cm^−1^): 3285, 1639, 1585, 1530, 1514, 1487, 1435, 1393, 1302, 1271, 1249, 1210, 1073, 1010, 963, 812, 739; ^1^H-NMR (DMSO-*d_6_*), δ: 10.54 (s, 1H), 10.17 (br. s, 1H), 7.88–7.84 (m, 2H), 7.79 (d, 2H, *J* = 8.5 Hz), 7.66 (d, 1H, *J* = 8.3 Hz), 7.54 (d, 2H, *J* = 8.5 Hz), 7.46 (ddd, 1H, *J* = 8.0 Hz, *J* = 7.0 Hz, *J* = 1.3 Hz), 7.33 (ddd, 1H, *J* = 8.0 Hz, *J* = 7.0 Hz, *J* = 1.3 Hz), 7.25 (d, 1H, *J* = 8.5 Hz); ^13^C-NMR (DMSO-*d_6_*), δ: 165.92, 151.70, 139.00, 131.55, 131.34, 130.32, 128.02, 127.38, 127.08, 123.34, 123.07, 121.18, 118.35, 118.31, 114.86; HR-MS: for C_17_H_12_NO_2_Br [M+H]^+^ calculated 342.0124 *m*/*z*, found 342.0133 *m*/*z*.

*2-Hydroxy-N-(2-trifluoromethylphenyl)naphthalene-1-carboxamide* (**7a**). Yield 92%; Mp 124–125 °C; IR (cm^−1^): 3235, 1626, 1615, 1579, 1515, 1459, 1312, 1280, 1247, 1198, 1162, 1107, 1061, 1035, 825, 756; ^1^H-NMR (DMSO-*d_6_*), δ: 10.32 (br. s), 10.05 (s, 1H), 7.93–7.76 (m, 6H), 7.52–7.48 (m, 2H), 7.34 (ddd, 1H, *J* = 8.0 Hz, *J* = 7.0 Hz, *J* = 1.3 Hz), 7.26 (d, 1H, *J* = 9.0 Hz); ^13^C-NMR (DMSO-*d_6_*), δ: 166.86, 152.29, 135.83 (q, *J* = 2.2 Hz), 133.10, 131.59, 130.64, 129.91, 128.02, 127.51, 126.94, 126.62, 126.37 (q, *J* = 5.1 Hz), 124.70 (q, *J* = 29.3 Hz), 123.69 (q, *J* = 272.9 Hz), 123.59, 123.03, 118.29, 117.25; HR-MS: for C_18_H_12_NO_2_F_3_ [M+H]^+^ calculated 332.0893 *m*/*z*, found 332.0901 *m*/*z*.

*2-Hydroxy-N-(3-trifluoromethylphenyl)naphthalene-1-carboxamide* (**7b**). Yield 72%; Mp 153–156 °C; IR (cm^−1^): 3296, 3060, 1660, 1651, 1622, 1583, 1548, 1541, 1450, 1435, 1332, 1240, 1205, 1157, 1116, 816, 792, 753, 739, 693; ^1^H-NMR (DMSO-*d_6_*), δ: 10.77 (s, 1H), 10.22 (br. s, 1H), 8.38 (s, 1H), 7.97 (d, 1H, *J* = 8.3 Hz), 7.90–7.85 (m, 2H), 7.70 (d, 1H, *J* = 8.5 Hz), 7.62–7.58 (m, 1H), 7.49–7.45 (m, 2H), 7.34 (ddd, 1H, *J* = 8.0, *J* = 7.0, *J* = 1.3 Hz), 7.27 (d, 1H, *J* = 8.8 Hz); ^13^C-NMR (DMSO-*d_6_*), δ: 166.35, 151.82, 140.36, 131.31, 130.49, 130.00, 129.52 (q, *J* = 31.6 Hz), 128.03, 127.39, 127.16, 124.22 (q, *J* = 272.9 Hz), 123.32, 123.12, 122.78, 119.67 (q, *J* = 3.7 Hz), 118.35, 118.06, 115.26 (q, *J* = 3.7 Hz); HR-MS: for C_18_H_12_NO_2_F_3_ [M+H]^+^ calculated 332.0893 *m*/*z*, found 332.0900 *m*/*z*.

*2-Hydroxy-N-(4-trifluoromethylphenyl)naphthalene-1-carboxamide* (**7c**). Yield 70%; Mp 216–218 °C; IR (cm^−1^): 3267, 3053, 1660, 1631, 1598, 1583, 1513, 1409, 1322, 1198, 1116, 1108, 1067, 1017, 836, 813, 800, 752; ^1^H-NMR (DMSO-*d_6_*), δ: 10.79 (s, 1H), 10.22 (s, 1H), 8.03 (d, 2H, *J* = 8.5 Hz), 7.90–7.85 (m, 2H), 7.74 (d, 2H, *J* = 8.5 Hz), 7.68 (d, 1H, *J* = 8.3 Hz), 7.47 (ddd, 1H, *J* = 8.0 Hz, *J* = 7.0 Hz, *J* = 1.3 Hz), 7.34 (ddd, 1H, *J* = 8.0 Hz, *J* = 7.0 Hz, *J* = 1.3 Hz), 7.27 (d, 2H, *J* = 9.0 Hz); ^13^C-NMR (DMSO-*d_6_*), δ: 166.40, 151.82, 143.15, 131.30, 130.49, 128.05, 127.39, 127.16, 126.09 (q, *J* = 3.67 Hz), 124.51 (q, *J* = 263.4 Hz), 123.36 (q, *J* = 31.5 Hz), 123.35, 123.12, 119.16, 118.35, 118.09; HR-MS: for C_18_H_12_NO_2_F_3_ [M+H]^+^ calculated 332.0893 *m*/*z*, found 332.0900 *m*/*z*.

*2-Hydroxy-N-(2-nitrophenyl)naphthalene-1-carboxamide* (**8a**). Yield 73%; Mp 139–141 °C; IR (cm^−1^): 3329, 1651, 1634, 1578, 1496, 1462, 1428, 1341, 1269, 1195, 1143, 823, 790, 735; ^1^H-NMR (DMSO-*d_6_*), δ: 10.35 (s, 1H), 10.12 (s, 1H), 8.03 (d, 1H, *J* = 9.3 Hz), 7.89–7.80 (m, 4H), 7.69 (d, 1H, *J* = 8.5 Hz), 7.53–7.50 (m, 1H), 7.46 (ddd, 1H, *J* = 8.0 Hz, *J* = 7.0 Hz, *J* = 1.3 Hz), 7.32 (ddd, 1H, *J* = 8.0 Hz, *J* = 7.0 Hz, *J* = 1.3 Hz), 7.25 (d, 1H, *J* = 9.0 Hz); ^13^C-NMR (DMSO-*d_6_*), δ: 166.80, 152.30, 143.06, 133.12, 131.56, 130.60, 129.70, 128.00, 127.36, 127.11, 125.73, 124.85, 124.56, 123.56, 123.04, 118.30, 117.36; HR-MS: for C_17_H_12_N_2_O_4_ [M+H]^+^ calculated 309.0870 *m*/*z*, found 309.0877 *m*/*z*.

*2-Hydroxy-N-(3-nitrophenyl)naphthalene-1-carboxamide* (**8b**). Yield 60%; Mp 255–257 °C; IR (cm^−1^): 3403, 2837, 2539, 1629, 1601, 1571, 1455, 1429, 1304, 1248, 1206, 1167, 1149, 1089, 911, 794, 760, 721; ^1^H-NMR (DMSO-*d_6_*), δ: 10.88 (s, 1H), 10.41 (s, 1H), 8.03 (dd, 1H, *J* = 8.3, *J* = 1.5 Hz), 7.95 (d, 1H, *J* = 8.5 Hz), 7.92–7.85 (m, 3H), 7.77 (ddd, 1H, *J* = 8.3 Hz, *J* = 7.0 Hz, *J* = 1.3 Hz), 7.51 (ddd, 1H, *J* = 8.0 Hz, *J* = 7.0 Hz, *J* = 1.3 Hz), 7.41 (ddd, 1H, *J* = 8.3 Hz, *J* = 7.0 Hz, *J* = 1.3 Hz), 7.36 (ddd, 1H, *J* = 8.0 Hz, *J* = 7.0 Hz, *J* = 1.3 Hz), 7.26 (d, 1H, *J* = 8.8 Hz); ^13^C-NMR (DMSO-*d_6_*), δ: 165.79, 152.49, 142.11, 134.13, 131.72, 131.54, 131.22, 128.07, 127.51, 127.17, 125.10, 125.01, 124.92, 123.54, 123.23, 118.25, 116.45; HR-MS: for C_17_H_12_N_2_O_4_ [M+H]^+^ calculated 309.0870 *m*/*z*, found 309.0874 *m*/*z*.

*2-Hydroxy-N-(4-nitrophenyl)naphthalene-1-carboxamide* (**8c**). Yield 81%; Mp 195–197 °C; IR (cm^−1^): 3345, 1648, 1614, 1552, 1506, 1435, 1329, 1300, 1257, 1212, 1176, 1111, 960, 895, 847, 808, 748, 738; ^1^H-NMR (DMSO-*d_6_*), δ: 11.05 (s, 1H), 10.31 (br. s, 1H), 8.29 (d, 2H, *J* = 8.3 Hz), 8.05 (d, 2H, *J* = 8.3 Hz), 7.92–7.86 (m, 2H), 7.67 (d, 1H, *J* = 8.3 Hz), 7.48 (ddd, 1H, *J* = 8.0 Hz, *J* = 7.0 Hz, *J* = 1.3 Hz), 7.34 (ddd, 1H, *J* = 8.0 Hz, *J* = 7.0 Hz, *J* = 1.3 Hz), 7.27 (d, 1H, *J* = 8.3 Hz); ^13^C-NMR (DMSO-*d_6_*), δ: 166.73, 152.00, 145.72, 142.31, 131.23, 130.77, 128.13, 127.39, 127.31, 125.09, 123.21, 123.16, 119.02, 119.00, 118.33; HR-MS: for C_17_H_12_N_2_O_4_ [M+H]^+^ calculated 309.0870 *m*/*z*, found 309.0876 *m*/*z*.

### 3.3. Lipophilicity Determination by HPLC (Capacity Factor k/Calculated log k)

A HPLC system Agilent 1200 equipped with DAD detector (Agilent, Santa Clara, CA, USA) was used. A chromatographic column Symmetry^®^ C_18_ 5 μm, 4.6 × 250 mm, Part No. WAT054275, (Waters Corp., Milford, MA, USA) was used. The HPLC separation process was monitored and evaluated by EZChrom Elite software ver. 3.3.2 (Agilent). Isocratic elution by a mixture of MeOH p.a. (60%) and H_2_O-HPLC Mili-Q grade (40%) as a mobile phase was used. The total flow of the column was 1.0 mL/min, injection 20 μL, column temperature 40 °C and sample temperature 10 °C. The detection wavelength 210 nm was chosen. The KI methanolic solution was used for the dead time (t_D_) determination. Retention times (t_R_) were measured in minutes. The capacity factors *k* were calculated according to formula *k* = (t_R_ − t_D_)/t_D_, where t_R_ is the retention time of the solute, whereas t_D_ denotes the dead time obtained using an unretained analyte. Log *k*, calculated from the capacity factor *k*, is used as the lipophilicity index converted to log *P* scale. The log *k* values of the individual compounds are shown in Table 1.

### 3.4. Study of Inhibition of Photosynthetic Electron Transport (PET) in Spinach Chloroplasts

Chloroplasts were prepared from spinach (*Spinacia* *oleracea* L.) according to Masarovicova and Kralova [56]. The inhibition of photosynthetic electron transport (PET) in spinach chloroplasts was determined spectrophotometrically (Genesys 6, Thermo Electron Scientific Instruments, Madison, WI, USA), using an artificial electron acceptor 2,6-dichlorophenol-indophenol (DCPIP) according to Kralova *et al.* [57], and the rate of photosynthetic electron transport was monitored as a photoreduction of DCPIP. The measurements were carried out in phosphate buffer (0.02 mol/L, pH 7.2) containing sucrose (0.4 mol/L), MgCl_2_ (0.005 mol/L) and NaCl (0.015 mol/L). The chlorophyll content was 30 mg/L in these experiments and the samples were irradiated (~100 W/m^2^ with 10 cm distance) with a halogen lamp (250 W) using a 4 cm water filter to prevent warming of the samples (suspension temperature 22 °C). The studied compounds were dissolved in DMSO due to their limited water solubility. The applied DMSO concentration (up to 4%) did not affect the photochemical activity in spinach chloroplasts. The inhibitory efficiency of the studied compounds was expressed by IC_50_ values, *i.e.*, by molar concentration of the compounds causing 50% decrease in the oxygen evolution rate relative to the untreated control. The comparable IC_50_ value for a selective herbicide 3-(3,4-dichlorophenyl)-1,1-dimethylurea, DCMU (Diurone^®^) was about 1.9 μmol/L. The results are summarized in Table 1.

### 3.5. Study of Chlorophyll a and Aromatic Amino Acids Fluorescence in Spinach Chloroplasts

The fluorescence emission spectra of chlorophyll *a* (Chl*a*) and aromatic amino acids (AAA) in spinach chloroplasts were recorded on fluorescence spectrophotometer F-2000 (Hitachi, Tokyo, Japan) using excitation wavelength λ_ex_ = 436 nm for monitoring fluorescence of Chl*a* and λ_ex_ = 275 nm for monitoring AAA fluorescence, excitation slit 20 nm and emission slit 10 nm. The samples were kept in the dark for 2 min before measuring. The phosphate buffer used for dilution of the chloroplast suspension was the same as described above. Due to low aqueous solubility the compounds were added to chloroplast suspension in DMSO solution. The DMSO concentration in all samples was the same as in the control (10%). The chlorophyll concentration in chloroplast suspension was 10 mg/L.

### 3.6. *In Vitro* Antibacterial Susceptibility Testing

The synthesized compounds were evaluated for *in vitro* antibacterial activity against representatives of multidrug-resistant bacteria, clinical isolates of methicillin-resistant *Staphylococcus aureus* (MRSA) 63718, SA 630 and SA 3202 that were obtained from the National Institute of Public Health, Prague, Czech Republic. *Staphylococcus aureus* ATCC 29213 was used as a reference and quality control strain. Ampicillin (Sigma-Aldrich) was used as the standard. Prior to testing, each strain was passaged onto nutrient agar (Oxoid, Hampshire, UK) with 5% of bovine blood, and bacterial inocula were prepared by suspending a small portion of bacterial colony in sterile phosphate buffered saline (pH 7.2–7.3). The cell density was adjusted to 0.5 McFarland units using a densitometer (Densi-La-Meter, LIAP, Riga, Latvia). The final inoculum was made by 1:20 dilution of the suspension with the Mueller-Hinton broth (MH broth). The compounds were dissolved in DMSO (Sigma), and the final concentration of DMSO in the MH broth (Oxoid) did not exceed 2.5% of the total solution composition. The final concentrations of the evaluated compounds ranging from 256 μg/mL to 0.008 μg/mL. The broth dilution micro-method modified according to NCCLS guidelines [58,59] in MH broth was used to determine the minimum inhibitory concentration (MIC). Drug-free controls, sterility controls and controls consisted of MH broth and DMSO alone were included. The determination of results was performed visually after 24 h of static incubation in the darkness at 37 °C in an aerobic atmosphere. The MICs were defined as the lowest concentration of the compound at which no visible bacterial growth was observed. The results are summarized in Table 1.

### 3.7. *In Vitro* Antimycobacterial Evaluation

The evaluation of *in vitro* antimycobacterial activity of the compounds was performed against *Mycobacterium marinum* CAMP 5644, *M. kansasii* DSM 44162 and *M. smegmatis* ATCC 700084. The broth dilution micro-method in Middlebrook 7H9 medium (Difco, Lawrence, KS, USA) supplemented with BD BBL™ Middlebrook ADC Enrichment (Becton, Dickinson & Comp., Franklin Lakes, NJ, USA) was used to determine the minimum inhibitory concentration (MIC) as previously described [60]. The compounds were dissolved in DMSO (Sigma-Aldrich), and the final concentration of DMSO did not exceed 2.5% of the total solution composition. The final concentrations of the evaluated compounds ranging from 256 μg/mL to 0.125 μg/mL were obtained by twofold serial dilution of the stock solution in microtiter plate with sterile medium. Bacterial inocula were prepared by transferring colonies from culture to sterile water. The cell density was adjusted to 0.5 McFarland units using a densitometer (Densi-La-Meter, LIAP, Riga, Latvia). The final inoculum was made by 1:1000 dilution of the suspension with sterile water. Drug-free controls, sterility controls and controls consisted of medium and DMSO alone were included. Results were recorded visually after 3 days of static incubation in the darkness at 37 °C aerobically for *M. smegmatis*, after 7 days of static incubation in the darkness at 37 °C aerobically for *M. kansasii* and after 21 days of static incubation in the darkness at 28 °C aerobically for *M. marinum*.

A well characterised clinical isolate of *Mycobacterium avium* subsp. *paratuberculosis* (CIT03) was grown in Middlebrook broth (MB), supplemented with Oleic-Albumin-Dextrose-Catalase supplement (OADC, Becton, Dickinson & Comp., Franklin Lakes, NJ, USA) and mycobactin J (2 µg/mL). Identification of this isolate was performed using biochemical and molecular protocols. At log phase growth, a culture sample (10 mL) was centrifuged at 15,000 rpm/20 min using a bench top centrifuge (Model CR 4-12, Jouan Inc., Winchester, VA, USA). Following removal of the supernatant, the pellet was washed in fresh Middlebrook 7H9GC broth and re-suspended in fresh supplemented MB (10 mL). The turbidity was adjusted to match McFarland standard No. 1 (3 × 10^8^ cfu) with MB broth. A further 1:20 dilution of the culture was then performed in MB broth. The antimicrobial susceptibility of the mycobacterial species was investigated in a 96-well plate format. In these experiments, sterile deionised water (300 µL) was added to all outer-perimeter wells of the plates to minimize evaporation of the medium in the test wells during incubation. Each evaluated compound (100 µL) was incubated with each of the mycobacterial species (100 µL). Dilutions of each compound were prepared in duplicate. For all synthesized compounds, final concentrations ranged from 1,000 µg/mL to 8 µg/mL. All compounds were prepared in DMSO and subsequent dilutions were made in supplemented MB. The plates were sealed with parafilm and incubated at 37 °C, for 11 days in the case of *M. avium paratuberculosis*. Following incubation, a 10% addition of alamarBlue (AbD Serotec, Kidlington, UK) was mixed into each well and readings at 570 nm and 600 nm were taken, initially for background subtraction and subsequently after 24 h re-incubation. The background subtraction is necessary for strongly coloured compounds, where the colour may interfere with the interpretation of any colour change. For non-interfering compounds, a blue colour in the well was interpreted as an absence of growth and a pink colour was scored as growth. The minimum inhibitory concentrations (MICs) were initially defined as the lowest concentration which prevented a visual colour change from blue to pink.

The MICs were defined as the lowest concentration of the compound at which no visible bacterial growth was observed. The MIC value is routinely and widely used in bacterial assays and is a standard detection limit according to the Clinical and Laboratory Standards Institute (CLSI). Isoniazid (Sigma-Aldrich) was used as reference antimycobacterial drug. The results are summarized in Table 1.

### 3.8. *In Vitro* Cytotoxicity Assay

Human monocytic leukemia THP-1 cells were obtained from the European Collection of Cell Cultures (ECACC, Salisbury, UK; Methods of characterization: DNA Fingerprinting (Multilocus probes) and isoenzyme analysis). These cells were routinely cultured in RPMI 1640 (Lonza, Verviers, Belgium) medium supplemented with 10% fetal bovine serum (FBS, Sigma-Aldrich), 2% l-glutamine, 1% penicillin and streptomycin (Lonza, Verviers, Belgium) at 37 °C with 5% CO_2_. Cells were passaged at approximately 1 week intervals. Cells were routinely tested for the absence of mycoplasma (Hoechst 33258 staining method). The tested compounds were dissolved in DMSO (Sigma-Aldrich) and added in five increasing concentrations to the cell suspension in the culture medium. The maximum concentration of DMSO in the assays never exceeded 0.1%. Subsequently, the cells were incubated for 24 h at 37 °C with 5% CO_2_ to various compound concentrations ranging from 0.37 to 20 μmol/L in RPMI 1640 medium. Cell toxicity was determined using a Cytotoxicity Detection Kit^PLUS^ Lactate dehydrogenase (LDH) assay kit (Roche Diagnostics, Mannheim, Germany) according to the manufacturer’s instructions, as described previously [11,26,27]. For LDH assays, cells were seeded into 96-well plates (5 × 10^4^ cells·well^−1^ in 100 μL culture medium) in triplicate in serum-free RPMI 1640 medium, and measurements at 492 nm wavelength (Synergy 2 Multi-Mode Microplate Reader, BioTek, Winooski, VT, USA) were taken 24 h after the treatment with tested compounds. The median lethal dose values, LD_50_, were deduced through the production of a dose-response curve. All data were evaluated using GraphPad Prism 5.00 software (GraphPad Software, San Diego, CA, USA). The results are summarized in Table 1.

## 4. Conclusions

A series of twenty-two 2-hydroxy-*N*-phenylnaphthalene-1-carboxanilides were prepared by means of microwave synthesis and subsequently characterized. All the compounds were tested for their ability to inhibit photosynthetic electron transport (PET) in spinach chloroplasts (*Spinacia* *oleracea* L.), and for their *in vitro* antimicrobial activity against *S. aureus*, three methicillin-resistant *S. aureus* strains, *M. marinum*, *M. kasasii*, *M. smegmatis*. and a clinical isolate of *M. avium paratuberculosis.* The *ortho*-substituted compounds showed lower biological activity than the *meta-* and *para*-substituted ones, therefore it can be concluded that, *meta*- or *para*-position of substituents on anilide is essential for biological effect. Furthermore, it can be stated that the dependence of activity on the lipophilicity was parabolic or bilinear and mostly the dependence of activity on electron parameters of individual substituents increased with electron-withdrawing properties that also influenced *in vitro* cytotoxicity against THP-1 cells. In conclusion it can be stated that unsubstituted **1**, **8a** (R = 2-NO_2_) and **8b** (R = 3-NO_2_) can be considered as non-toxic lead compounds for the subsequent design of novel antimicrobial agents.
